# Effect of Artificial Aging Protocols on Surface Gloss of Resin Composites

**DOI:** 10.1155/2017/3483171

**Published:** 2017-05-22

**Authors:** Rafael Santos Rocha, Amanda Carvalho Oliveira, Taciana Marco Ferraz Caneppele, Eduardo Bresciani

**Affiliations:** Department of Restorative Dentistry, São Paulo State University (UNESP), Institute of Science and Technology, São José dos Campos, SP, Brazil

## Abstract

The purpose of this study was to evaluate the effect of aging protocols on surface gloss of composites. Cylindrical resin composite specimens (6 mm in diameter, 1 mm thick) were fabricated and divided into three groups (*N* = 60): microfilled (MiFi), nanohybrid (NaHy), and nanofilled (NaFi). Specimens were distributed into four aging subgroups: thermocycling (5° to 55°C, 15,000 cycles); ethanol immersion (15 days); brushing (10,750 cycles); and light aging (216 h). Surface gloss readings (Novo-Curve, Rhopoint TM, England) were performed at baseline (R0) and after every one-third of aging protocols (R1 to R3). Data were submitted to one-way repeated measures ANOVA and Tukey's test (5%). Overall, surface gloss alterations were detected over time (*p* < 0.001). Thermocycling reduced surface gloss, except for NaHy. Ethanol immersion resulted in surface gloss reduction after R1 for MiFi and NaFi, while reduction after R1 and R2 was detected for NaHy. For brushing, gloss reduction was detected after R1 and R3 for all composites. For light aging, gloss was reduced after R1 and R2 for MiFi and NaFi, while a reduction only after R1 was detected for NaHy. The studied aging protocols affect surface gloss differently, being material and aging therapy dependent. In general, the surface gloss is reduced with aging.

## 1. Introduction

The demand of patients for esthetic treatments boosted the development of restorative materials simulating optical characteristics of natural teeth, allowing tooth characteristics to be mimicked with esthetically satisfactory results. On the other hand, problems related to the longevity of direct restorative treatments are still observed [1]. The major cause for restoration substitution in anterior teeth is related to esthetic reasons [1].

Among the esthetic factors, the maintenance of surface gloss is of great importance [2, 3]. This characteristic is usually related to the deterioration or wear of materials [4, 5]. This leads to unsuitable optical properties of restorations and to the necessity for repolishing, repairing, or restoration replacement [6].

It is known that composites undergo degradation in the oral environment; thus, studies analyze “in vitro” the effect of artificial aging on the mechanical and optical properties of those materials. Several types of aging have been suggested to simulate situations to which restorative materials are subjected, while in clinical service [4, 5]. The most frequently used aging protocols are thermocycling, immersion in liquid media such as ethanol or water, brushing simulation, and light aging [4, 5].

It is important to highlight that different types of composites might behave differently against aging stimuli [7–12]. Few studies have verified the influence of aging processes on the surface gloss of composites. The reported studies often evaluated the repolishing effectiveness [13, 14] or the brushing influence on restorative materials' gloss [4, 5, 7, 8, 15, 16].

Due to the importance of the surface gloss of restorations within the esthetic clinical parameters, often requested by patients, it is important to assess how different aging methods would interfere in the composite's behavior. The aim of this study was to evaluate the effect of different types of resin composites and the degree of aging on their surface gloss. The null hypothesis of this study was that the tested aging protocol does not influence the surface gloss of resin composites.

## 2. Materials and Methods

### 2.1. Specimen Preparation

Three types of resins were used: microfilled (MiFi), nanohybrid (NaHy), and nanofilled (NaFi) (Table 1).

In total, sixty samples of each resin were fabricated using a stainless steel matrix 6 mm in diameter and 1 mm in thickness. The material was inserted in a single increment and a mylar strip and glass slide were positioned over the resin. Specimen was light cured for 40 s using a LED device (Radii Cal Curing Light, SDI, Victoria, Australia) with 900 mW/cm^2^ in irradiance, measured by a radiometer (Demetron LED radiometer, Kerr Corporation, Middletown, WI, USA). The specimen was attached to a metal holder and polished with sequential abrasive papers (600 to 1200 Grit) in a polishing device (DP-10, Panambra Industrial e Técnica AS, Sao Paulo, SP, Brazil), for 30 s in the first two abrasive papers (600 and 800 Grit), and for 120 s in the final abrasive paper (1200 Grit).

### 2.2. Surface Gloss Analysis

The gloss reading was performed using a Novo-Curve device (Rhopoint TM, East Sussex, England) with a 2 mm × 2 mm area and a 60° geometry (light incidence), with values expressed in Gloss Units (GU) [9–11]. A metal screen was used to eliminate the interference of environmental light. Three randomized measurements were performed for each sample during each evaluation stage, and the average of those measurements was used for the statistical analysis.

### 2.3. Experimental Design

**Figure 1:**
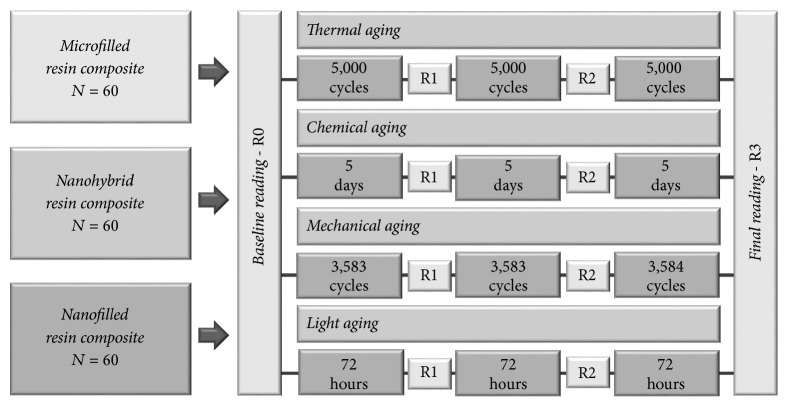
Schematic diagram of experimental design of this study.

The baseline surface gloss reading (R0) was performed after the specimens' fabrication and polishing. After R0, samples of each type of resin were randomly divided into four subgroups, according to the type of aging. Two independent readings were conducted in intermediate phases during aging (R1 and R2), and the final reading (R3) was performed at the end of the aging protocols.  Figure 1 presents the experimental design of the study.

### 2.4. Artificial Aging

#### 2.4.1. Thermal Aging

Specimens were submitted to 15,000 thermal cycling with the temperature at 5°C and 55°C and a 5 s dwell time. The thermocycler Erios (Erios, Sao Paulo, SP, Brazil) was employed. After every 5,000 cycles, specimens were removed from the device (5,000, 10,000, and 15,000 cycles), and a surface gloss reading was performed.

#### 2.4.2. Chemical Aging

Specimens were immersed in a 75% ethanol solution for 15 days. Each sample was placed in an individual plastic microtube containing 1 ml of the solution. Every 5 days, the sample was removed, the surface was dried with absorbent paper, and a surface gloss reading was performed (baseline, 5, 10, and 15 days). The ethanol solution was changed after each surface gloss reading (every 5 days).

#### 2.4.3. Mechanical Aging

Specimens were submitted to simulated brushing using MEV-2T equipment (Odeme Equipamentos Médicos e Odontológicos Ltda., Joaçaba, SC, Brazil). The toothpaste suspension was prepared by mixing 6 ml distilled water with 6 g of toothpaste (Colgate Total 12, Colgate-Palmolive, Sao Paulo, Brazil, RDA 70 *µ*m) [17]. A soft toothbrush was used during the experiments (Sanifill ultra professional, Sanifill, Sao Paulo, SP, Brazil). Brushing cycles consisted of 3.8 cm motion amplitude with a 200 g weight, totaling 10,750 cycles. The aging was performed in a controlled temperature at 37°C. After every 3,583 cycles the surface gloss was assessed. The toothbrush and toothpaste suspension were changed every reading period (R1, R2, and R3).

#### 2.4.4. Light Aging

Specimens were submitted to the aging device SUNTEST CPS+ (Atlas, Gelnhausen, Germany) using a xenon lamp, with exposure to filtered UV light, following the ISO 7491 standard. The parameters were the following: each cycle was composed of two hours at 55 ± 5°C and irradiation at 765 W/m^2^, followed by one hour at 37 ± 5°C and no light irradiation, totaling 3 hours. Every 72 hours (24 cycles) a gloss reading was performed. The total protocol (72 cycles, 216 hours) simulated an exposure of 160 klux, corresponding to intense natural light.

### 2.5. Statistics

The descriptive statistics are presented in GU average values and standard deviation. The one-way repeated measures ANOVA and multiple comparison test (Tukey's test) constituted the inferential statistic for each aging protocol and tested resin composite. The significance level was set at 5%.

## 3. Results

**Figure 2:**
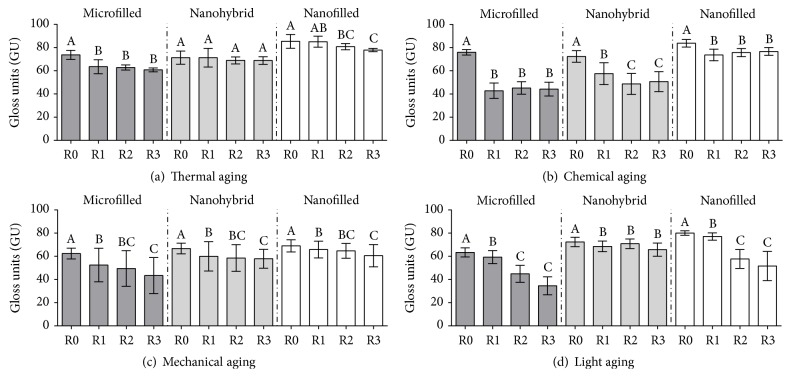
Comparison of surface gloss in relation to (a) thermal aging, (b) chemical aging, (c) mechanical aging, and (d) light aging. Different letters indicate statistical differences within each type of resin composite; *p* < 0.05.

The surface gloss results of the tested aging protocols and resin composites are presented in Figure 2.

In general, the tested resins behaved differently according to the different types of aging.

The percentage of surface gloss reduction in relation to the baseline reading is presented in Table 2.

### 3.1. Thermal Aging

One-way repeated measures ANOVA revealed differences in the GU after aging (*p* < 0.001), except for NaHy (*p* = 0.173). For the MiFi group, the surface gloss decreased in R1 and was stable after that reading period. Thermocycling did not interfere in the gloss of the NaHy resins. For the NaFi resins, the gloss decreased in R2 in comparison to R0 and also decreased in R3 in comparison to R1, with similar gloss values between R0 and R1, and R2 and R3 (Figure 2(a)).

The greatest gloss reduction of 17.5% was detected for MiFi.

### 3.2. Chemical Aging

One-way repeated measures ANOVA revealed differences in the GU after immersion in ethanol for all tested resin composites (*p* < 0.001). For the MiFi and NaFi groups, the surface gloss decreased only at the first immersion time, with no further reduction with time (R0 > R1 = R2 = R3). The NaHy resin presented a gloss reduction for R1 and R2, stabilizing after R2 (R0 > R1 > R2 = R3) (Figure 2(b)).

The greatest gloss reduction of 44.4% was detected for the MiFi resin.

### 3.3. Mechanical Aging

One-way repeated measures ANOVA showed differences in the GU through time for all the tested resins (*p* < 0.001). The brushing time negatively influenced the surface gloss over time (R0 > R1 > R3, being R1 = R2 and R2 = R3 for all tested resins) (Figure 2(c)). All tested resin composites presented a similar pattern of surface gloss reduction while under tooth brushing.

The greatest gloss reduction was detected for the MiFi resin at R3 in comparison to R0, the reduction being 30.3%.

### 3.4. Light Aging

One-way repeated measures ANOVA revealed differences in the GU through time for all the tested resins (*p* < 0.001). The surface gloss decreased in R1 and stabilized after R2 in the MiFi and NaFi groups. For the NaHy group, surface gloss reduction occurred only for R1 with no further reduction for the following aging periods (Figure 2(d)).

The greatest gloss reduction was detected for the MiFi resin at R3 in comparison to R0, the reduction being 44.3%.

## 4. Discussion

The tested null hypothesis, within each aging group, was rejected. The single exception was detected for NaHy under thermal aging, in which no alteration of the surface gloss was detected.

In case surface gloss was reduced, different patterns of reduction were noticed. The most common patterns were a reduction at R1 with subsequent stabilization or gradual pattern of gloss reduction over time.

Specimens submitted to thermocycling presented a reduction of gloss over time. It is known that thermal cycling might create internal tensions in the resin structure due to differences in the linear thermal expansion coefficient of the organic matrix and filler components, leading to degradation [18] and possible surface microcracks [19]. Reports on the organic matrix degradation also explain the possible surface deterioration of resin composites under thermal stresses [18–21]. The presence of water is another factor to be considered, as it might lead to degradation of the silane layer between the filler and organic matrix or even lead to water sorption by the resin [22]. The present surface degradation resulting from thermal aging was possibly sufficient to interfere with the gloss properties. Thermocycling is reported to interfere with the surface roughness of resin composites [23], and since surface roughness is related to a reduced surface gloss, it sounds plausible to assume that thermal aging will possibly lead to surface gloss reduction, as found for NaFi and MiFi (Table 2 and Figure 2). The NaHy group did not undergo surface gloss alteration over time and possibly had less surface degradation compared to the other tested resin composites.

Although the behavior of NaFi was different from NaHy, the percentage of surface gloss alteration (Table 2) followed the same pattern for both resins. One should consider the clinical implications of such alterations for indicating or not such resin composites.

Also regarding thermal aging, the NaFi group presented a higher surface gloss. The literature points out that small particles in resins possibly decrease the diffuse reflection of materials, which results in a visually glossier surface [24]. The spherical-sized particles in the NaFi group might also be a factor of a higher reflection when compared to the irregular-sized fillers in the NaHy group [12].

Regarding chemical aging, it is reported that differences in inorganic fillers influence the diffusion of aqueous solutions and ethanol [25], resulting in different aging patterns. This observation about the influence of resin composition regarding aging agent penetration [26] probably explains the differences found among the tested resins. In a study reporting mechanical property changes [27], a great alteration was detected on mechanical properties with ethanol aging. In that study, along with the organic matrix degradation [28, 29], the degradation of the ester bonds [26, 30] and the silane agent were also discussed as being responsible for the deterioration of the mechanical properties when submitted to ethanol aging. A study [31] reported on the influence of light-curing protocols and resin immersion in ethanol resulted in the polymer softening, due to the dramatic polymer swelling and the consequent weakening of the polymeric chains' cohesive forces [32]. In that study, the lower cross-link density resulted in a greater surface degradation. Although light-curing was standardized in the current study, differences in the cross-link density of different resin composites associated with different possible patterns of ethanol infiltration might explain the present results, in which two resins (MiFi and NaFi) presented the greatest gloss alterations after the first aging cycle, while NaHy also presented a surface gloss alteration after the second aging cycle (Table 2 and Figure 2).

Regarding brushing, the pattern of surface gloss reduction was similar within the tested types of resin (Table 2 and Figure 2). Despite initial polishing procedures having been standardized, it is known that resin wear depends on its inorganic and organic components. The NaFi resin presented a higher numerical surface gloss, a fact supported by improved polishing properties of nanotechnology resins [33]. On the other hand, the worst performance for microfilled resin might be related to a less rigid organic portion, resulting in protrusion of fillers or spaces without inorganic filler after the polishing/brushing procedure [33].

The literature shows that brushing produces microscopic and macroscopic irregularities, resulting in a diffused reflection of the incident light, reducing surface gloss [2, 34]. The present gloss reduction behavior is supported by other studies [12, 35], in which wear and progressively increased roughness are related to the increased number of cycles for simulated brushing. The abrasiveness of the toothpaste employed should also be pointed out. The RDA of Colgate 12, the toothpaste used in this study, is close to 70 *µ*m. There are toothpastes available with greater abrasiveness [17], and if they were used in the present study the behavior of the microfilled resin composites might have been worse or would have presented a different pattern due to their less rigid organic matrix, as previously discussed.

Interestingly, brushing did not result in the greatest percent of gloss reduction among the resins, highlighting the importance of understanding other aging processes' influence over the surface gloss of dental materials. It is believed that surface polishing is strongly related to surface gloss; however, the present data shows other aging protocols, like ethanol immersion, might have a greater influence on surface gloss.

When submitted to light aging, the light exposure resulted in a gradual decrease of gloss over time. A similar study showed results that support the present observation [36]. The possible explanation is related to the fact that light aging degrades the organic portion of the resins, interfering with the light reflection of the inorganic fillers [37]. In a study of the effect of induced aging on the color of resins [38], the authors attributed the obtained results due to the superficial action of UV light as responsible for the material deterioration. This fact might be explained by a greater surface roughness detected in a study in which resin composites were aged under light protocols [39]. Surface deterioration might also explain the results of the gradual gloss reduction found in the present study.

Although the differences of the surface gloss alteration were detected among the tested resin composites under different aging protocols, the limit of the perceptibility and acceptability of surface gloss in clinical conditions has not been established yet. Thus, one of the limitations of the present study is related to the lack of information on the possible clinical correlation with the detected gloss alterations.

More studies should be conducted to complement the present obtained results. For extrapolating the results to clinic conditions, one should consider the presence of other aging agents and the possible lower intensity of the tested aging protocols in the oral environment.

## 5. Conclusions

Despite the limitations of this study, it was concluded that

- aging negatively affected the surface gloss, with no defined pattern according to the proposed aging cycles;
- tested composites presented different surface gloss, with the MiFi resins having the greatest percentage of surface gloss reduction for all the tested aging protocols; and
- surface gloss reduction is material and aging dependent, with NaHy presenting no surface gloss reduction during thermal aging.

## Figures and Tables

**Table 1:** Resin composites used in this study.

Resin composite	Manufacture	Color	Classification	Filler (wt.%)	Matrix
Microfiller-Durafill VS	HeraeusKulzer, Wehrheim, Germany	A2E	Microfiller	50.5	Bis-GMA, UDMA, TEGDMA
IPS Empress Direct	Ivoclar Vivadent, Amherst, NY, USA	A2E	Nanohybrid	78.1	UDMA, TCDMMA, Bis-GMA
Filtek Z350XT	3M-ESPE, St. Paul, MN, USA	A2E	Nanofiller	72.5	Bis-GMA, UDMA, -TEGDMA, Bis-EMA

**Table 2:** Percentage of surface gloss reduction after each reading (R1 to R3) in relation to R0.

Aging	Resin composite	R1	R2	R3
Thermal	MiFI	13.83%	14.79%	17.50%
	NaHy	0.06%	3.37%	3.51%
	NaFi	0.23%	5.28%	8.79%

Chemical	MiFI	43.61%	44.40%	41.77%
	NaHy	20.44%	32.73%	29.97%
	NaFi	12.17%	9.67%	8.59%

Mechanical	MiFI	15.73%	20.55%	30.34%
	NaHy	10.04%	12.29%	13.34%
	NaFi	4.78%	6.37%	12.45%

Light	MiFI	4.97%	28.04%	44.39%
	NaHy	5.25%	2.21%	9.95%
	NaFi	3.75%	27.97%	35.46%
